# Supplementary material for: Predictors of successful return to parkrun for first-time adult participants in Scotland
Source: PLOS Glob Public Health. 2023 Aug 16;3(8):e0001786. doi: 10.1371/journal.pgph.0001786 (PMC10431652; doi:10.1371/journal.pgph.0001786)
Supplement: Attachment — Submitted filename: Response to Reviewers.docx [file pgph.0001786.s001.docx]

PGPH-D-23-00368
Characteristics of parkrun That Encourage New Participants to Return
PLOS Global Public Health

I would like start by extending my gratitude to the two reviewers who have both provided extensive, insightful and constructive comments on the original draft of the manuscript. Their comments have substantially improved the quality manuscript.

Here’s the individual comments and my responses in italics below. I have accepted and acted upon all of the comments. The comments are in standard text and the responses in italics.

The structure of the Introduction is unclear. For example, it is unclear what parkrun is and why it is important. At present, the structure of the paper assumes insider knowledge. For example, where are these events located (e.g. line 65 needs context!). I think that the paper needs to start with an introduction to the growth in physical activity before moving to the barriers that individuals might face. You should also provide some explanation or evidence as to why you are indicating a connection between women and morbid obesity. I actually don’t think that you are suggesting this but the construction of the first paragraph indicates that this is the case!

*I have added much more detail about what parkrun is and where the events are located both specifically in terms of those used in this study and more widely. I’ve expanded the discussion of health issues associated with physical inactivity beyond obesity and removed any suggestion that there’s a gender bias in morbid obesity.*

No clear aim of the paper and/or research question is stated. You merely assert that you are applying a generalized linear model to the data – but why is this necessary?

*Another fair comment. A much clearer aim has been added focussing on knowledge gaps and the research questions the study is addressing.*

You have not really examined the literature to document what previous studies both within parkrun but also relating to exercise more broadly, have shown / told us. You assert that previous studies of parkrun have used descriptive statistics. This omits to recognise that qualitative studies have also been undertaken, for example, Warhurst & Black (2022) “Lost and found: parkrun, work and identity”, Qualitative Research in Sport, Exercise and Health <https://doi.org/10.1080/2159676X.2021.1924244>

*The introduction has been expanded to cover more of the parkrun literature and also more of the wider literature including the qualitative study mentioned by the reviewer which adds another very interesting layer to the expanding literature on the multifaceted benefits of parkrun.*

As indicated above, you have discerned only a very limited number of characteristics that you consider will encourage return without justification for the selection of these specifically. An extensive literature within health, psychology and sport/exercise has examined multiple other factors that have been shown to influence likelihood of exercise (and of parkrunning). For example, why have you collected data re water without considering other terrain that have been shown to enhance wellbeing such as open space generally within large urban environments (e.g Birch, J., Rishbeth, C., & Payne, S. R. (2020). Nature doesn't judge you–how urban nature supports young people's mental health and wellbeing in a diverse UK city. Health & Place, 62, 102296; also Martin, L., White, M. P., Hunt, A., Richardson, M., Pahl, S., & Burt, J. (2020). Nature contact, nature connectedness and associations with health, wellbeing and pro-environmental behaviours. Journal of Environmental Psychology, 68, 101389). There are a number of systematic reviews of this literature.

*I’ve made this clearer and also included more discussion about green and blue exercise and their benefits. The original focus was just on fresh and salt water as these were two landscape characteristics that could be quickly assessed on a presence/absence basis. Since submitting the manuscript we now have used GIS to quantify the land cover types within a 30m radius all the parkrun routes included in this study. The intention was to use these data in the development of another manuscript but in the light of the reviewers comments and the timing of its availability it has been included in this manuscript. The model of return rates has now been regenerated with the inclusion of measures of the proportion of the surrounding area on each parkrun route consisting of six land cover types (woodland, grassland, freshwater, saltwater, shore and urban). The outcomes of the model are consistent with the findings reported in the original version of the manuscript with land area covered by fresh water acting as a positive influence on return rates particularly for female participants. The increased land cover dataset also identified a positive influence of woodland on the return rate of participants.*

In the case of parkrun itself you have also not taken account of what has been shown to be a key factor in sustaining the parkrun community – that of the social aspects that surround the run such as the availability of a café or refreshments and/or post-run community gathering/s – see for example, Hindley, D. (2022). “More than just a run in the park”: an exploration of parkrun as a shared leisure space. Leisure Sciences, 42(1), 85-105; also Morris, P., & Scott, H. (2019). Not just a run in the park: a qualitative exploration of parkrun and mental health. Advances in mental health, 17(2), 110-123

*I have now added more reference to this important aspect of parkrun research and one that is relevant to this study.*

Methods: It is not clear from where you sourced the data that you analysed? You indicate that it is the parkrun results but someone without knowledge of parkrun won’t know what/where.

*More detail has been provided about where the data come from. I have also cited a paper which contains extensive details of how data of this type was generated for another parkrun dataset. This also contains full details of how elevation gain, surface type and remoteness were measured. I have added details about how each of these were calculated but this can be expanded further if reference to the extensive details in the published paper isn’t sufficient.*

Why only 56 events that data was extracted from? Why those 56? Were these just UK? Have you taken account of location more broadly?

*There was no sampling of specific parkruns. Every parkrun venue in Scotland that hosted an event during the study period was included. Each parkrun results page needs to been visited individually and then cut and pasted into an Excel Macro. Then new adult parkrunners need to be identified. Then I had to determine whether or not each of them had which involved accessing athlete history of all 20k+ individually. So, there was months of laborious work to generate the return rate data. It would be great to have extended the study to other regions of the UK and beyond but the time it would have taken to generate the dataset was prohibitive. The decision was taken to concentrate on a single country with a variety of parkrun event venue location types and use that as model system. Hence this study, like the published study referred to above, is limited to Scotland.*

Within the findings, a number of assertions are made that are unclear:
o (line 183/184) that “those that engaged more readily in physical activity were more likely to return” but what is meant by this as you do not have data on exercise readiness?

*This statement was referring to the Reece et al study. I’ve made that clearer by adding an additional citation the Reece study to this sentence as well.*

(lines 198-200) “Individuals who enjoyed the general experience of parkrun but maybe not the specific course at the venue they attended could be more likely to try a different parkrun if they have another local alternative”. What is your evidence for this assertion?

*The sentence is a logical interpretation of the finding mentioned above that proximity to another parkrun is positively associated with return rate. The sentence contains the word could and isn’t really that assertive but I’ve added in the phrase “One possible explanation..” to the beginning of the sentence.*

o (lines 203-204) “first-time participants with the slowest finishing times were more likely to attend smaller and more remote events”. Do you know this, or is it that smaller events typically have slower participants? – and perhaps there is a reason for this, for example, are they new events, remote events, specific terrain etc. etc.?

*I’ve decided to remove the second smaller model that determined this from the paper as I don’t think it really contributed very much to help make room for the more informative land cover analyses.*

• You do not interpret the data/findings is made. As one example, the first para suggests, that returning to Scottish parkruns had a higher likelihood than shown in the wider data but it isn’t clear why (needs explanation) and the implications of this – especially given the health data for Scotland as a whole (although whether the hard-to-get-to are actually ever parkrunning …??)

*More interpretation has been added to the Discussion. There are two issues that cloud the comparison. One study straddled lockdown and the other didn’t so we need to understand the impacts of lockdown before drawing conclusions from this finding. Secondly, the pre-lockdown study used data from the UK, Ireland and Australia without provided separate figures for each nation. So even though there is health data for all nations it’s again difficult to drawn firm conclusions about differences between nations as we only have a nation specific figure for one. It is definitely worthy to note the finding and to speculate that it hints at return rates might be higher in Scotland but I think anything else would be open to criticisms about overinterpretation. Additional interpretation has been added throughout the Discussion.*

• The implications asserted from the findings indicate that the author does not fully understand the context of parkrun and how/when new parkruns are established! The establishment of a parkrun depends upon the availability of an open space that facilitates a safe 5k route, the availability of a team of volunteers (typically relates to the socio-economic status of the area / social capital of the lead), the willingness of a landowner. New concepts / practices are asserted at this stage. For example, social prescribing is potentially really important for the whole paper.

*The reviewer is describing the process by which the majority of parkrun events got established in the UK. Although the study was conducted in the UK the findings are relevant globally. Even in the UK many events were not created via the process referred to. For example, Sport England provided funding for parkrun to establish 200 additional parkruns and the parkrun Research Group identified the optimal locations for these on the basis of the locations of existing parkruns and levels of social deprivation in areas lacking them. A citation to these studies has been added. So large numbers of events can be created via processes outside of the usual structures so identifying characteristics of potential locations is an important contribution to the parkrun literature.*

*Furthermore, there are fewer barriers in relation to core team/volunteering requirements in many countries compared to the UK. Many European countries operate parkruns without marshals which really reduces the number of volunteers required so there’s more scope for parkrun to drive location choices of events. parkrun Global recently published their 5-year plan including their ambition to double the number of events and participants over the next five years. They are likely already reaching saturation point in some areas in terms of relying on core teams to establish events. So, they will need to review their event creation processes. Their 5 year plan also talks extensively about minimising barriers to event creation which suggests parkrun regularly review their event creation processes to try to avoid them being too restrictive. So, I don’t see the event creation process as a single fixed mechanism but something that can, and indeed should, evolve.*

*Finally, I would add that these findings are relevant to any organiser of mass participation sporting events not just parkrun. Thanks for raising this as I’ve added some discussion about the wider implications of these findings.*

In addition to the more major concerns that I have, I feel that greater clarity is needed as follows:
• p.3 It needs to be clear that parkrun is a running event as the preceding sentence refers to cycle routes.

*I’ve added clarification about the nature of how participants can engage in parkrun. Cycle paths were mentioned specifically because there has been a disproportionate increase in them as a positive upstream factor in many countries including Scotland, certainly compared to new parks and parkrun routes often incorporate sections of cycle paths with several in Scotland using relatively new stretches of cycle path.*

• Further explanation of parkrun is also needed as the current description assumes insider knowledge of the events and the datasets. For example, do you mean that by 2020 over 30m runs had been completed? (and is the COVID pandemic actually a necessary statement?).

*This sentence has been clarified and I agree it’s not needed to mention the cause of the suspension here as it has been referred to previously.*

• Ensure that all assertions are evidenced – for example, line 68, previous studies – what previous studies?

*I’ve been through the manuscript to ensure all relevant citations are in place. In the specific case mentioned that line had already been rewritten as part of expanding the background information on parkrun and this section now contains many more citations and that specific line is no longer present.*

• Take care using the term “primary data source” (line 82) as this might lead the reader to think you are generating primary data (ie an empirical study)

*I have removed the term primary data to avoid any potential confusion. Thanks for pointing this out.*

Reviewer #2: Review
This paper presents secondary data analysis from the parkrun database. It focuses on adult parkrun participants in Scotland between 2019-2020 – looking particularly at new participants who return. The authors have presented a generalised linear mixed modelling analysis and found important factors that influence whether participants return. These factors are important considerations for parkrun in encouraging new participants to return and when planning new events. This is a well-written manuscript; the text is clear and easy to read. It is also relevant to the journal and would interest the reader. My main concern with the paper is in level of detail. A stronger rationale is needed for why the paper focuses on parkrun, more detail is needed in the methods to explain how “remoteness” and travelling time were determined and the wider implications of the findings (beyond direct implications for parkrun) could be considered as this will interest a wider readership.

Major issues with this paper:
1. I would like to see a stronger rationale/justification for why parkrun is being used as an example of a “positive upstream factor” in this paper. What role could parkrun play in the global obesity epidemic? – perhaps here draw upon the potential for parkrun as a social prescription offer by health practitioners. Is it just the obesity epidemic that parkrun is potential useful for? Why is it important to understand patterns of participation, what could we do with this information? Perhaps the authors could use the existing parkrun research evidence base more to support this justification.

*Many thanks for these comments. Much more detail has been provided about the benefits of parkrun including how it is used in social prescription.*

2. The methods section would benefit from detail around:
a. How remoteness of parkrun venues was determined
b. How travelling time to parkrun venues was determined

*Much more detail has been provided about where the data come from. I have also cited a paper which contains extensive details of how data of this type was generated for another parkrun dataset. This also contains full details of how elevation gain, surface type and remoteness were measured. I have added details about how each of these were calculated to the new version of the manuscript but this can be expanded further if reference to the extensive details in the published paper isn’t sufficient.*

3. Parkrun is used here as an example of a ‘positive upstream’ initiative that can have an important role in the obesity epidemic.

*The benefits of parkrun have been described in much more detail and expanded well beyond tackling the obesity epidemic.*

Minor issues
Please see below for issues related to each manuscript section.

Title
I think the title would benefit from extra detail about the methods and/or location, also that the focus is on adults - if title word limit allows. A suggestion below – just a suggestion!
Characteristics of parkrun That Encourage New Adult Participants to Return: Generalised linear mixed modelling of parkrun data from Scotland

*Thanks for this suggestion. I have edited it to take into account the fact that it is not a comprehensive review of characteristics that encourage return but the derivation of novel factors and added in the word adult as well. Thanks to the reviewer for their suggestion which has helped create a more informative title.*

Abstract
Abstract provides a succinct summary of the research. More detail of the methods would be beneficial. For example – how many parkrun events were analysed
Line 16: GLMM is used in the abstract – should be written in full.

*The Abstract has been expanded to provide more details including the number of events. GLMM has been written out in full.*

Introduction
General comments about Introduction: The introduction mentions the obesity epidemic, and parkrun’s role as a ‘positive upstream factor’ in this. Doing so might be reducing parkrun to a physical activity intervention only, when the research suggests it could be more than that (e.g., community initiative, social capital, wellbeing, volunteering). Is the obesity epidemic the only public health concern that parkrun could address?

*Much more detail has been provided about the benefits of parkrun and how multifaceted they are.*

Line 31-37: The first paragraph mentions physical activity and sport – with terms seemingly being used interchangeably. Is this intentional? It could be clear – perhaps with a definition of physical activity that encompasses sport and exercise.

*Thanks for this observation. Physical activity and sport are not the same thing and had been used interchangeably. I have removed the word sport and replaced it with physical activity throughout.*

Line 35: 1.4 billion – is that globally?

*Yes, that has been clarified.*

Line 45-46: could add that the 2,000 events are across 22 countries.

*Done*

Line 49: “covid-19 pandemic” is later referred to as “SARS-cov2 pandemic” (line 94) – consistency needed.

*The second reference to the pandemic was deemed superfluous by the other reviewer so only the 1^st^ reference now exists.*

Lines 45-55: Readers who are unfamiliar with parkrun will benefit from a much broader explanation of the “5km event”. For example, it’s a free event, hosted in public open spaces. It is important to explain that people can run, walk, jog or participate as volunteers. Given the focus on new participants, it might be good to explain how people register and what they do (e.g. with their personal barcode/ID number) to get a record of their completion time, and how this feeds into the parkrun dataset.

*Much more details about how participants can take part in parkrun have been added.*

Lines 59-62: Where was this study based?

*I have added in details of where this was based.*

Lines 63-65: More detail needed here to help justify the focus on parkrun. What does the previous research into parkrun say? How does parkrun improve mental health and wellbeing? You might want to consider citing the scoping review by Grunseit et al. here (cited later on in the manuscript).

*Much more detail has been provided about the previous research into the benefits pf parkrun.*

Line 71: mention of health practitioners prescribing parkrun could be expanded upon- perhaps helping to justify why this research is focussing on parkrun as a public health offer.

*This specific line has now been removed in the restructuring of the introduction but there is now a more extensive section on social prescription and the parkrun practice initiative.*
Line 67: a rationale is provided for why the analysis looks at parkrun venues with blue space, but there is no rationale for why the analysis segregates by freshwater and saltwater. Could this make a difference?

*This analysis has now been superseded by a more detailed measure of the land-cover types which has been expanded to include six different land-use types. Each of these land-cover types is likely to have different environmental characteristics, host different communities of animals and plants. They will also vary in a range of sensory stimuli a parkrun participant is exposed e.g. sights, sounds, smells. I think investigating what sensory stimuli are associated with the attendance behaviour of parkrunners could be really interesting and a discussion about this has been added to the Discussion. Thanks to the reviewer from prompting this.*

Methods
General comments about Methods: The methods section would benefit from more detail in places. It needs an explanation of how “remoteness” was calculated and also how travel time to the parkrun was determined. Why was travel time preferred over distance? (see point below).

*Details have been added in addition to a citation to a paper which used the same variables and has extensive details about how they were calculated.*

Line 87: adults - is this 18 years and over – please specify.

*Yes, this has been clarified in the methods.*

Line 94-95: the additional characteristics of parkrun venues – where was this information collected from? By who?

*More details about these characteristics have been added to the Methods.*

Line 103: I admit to being no expert in this analysis method. Is generalised linear mixed modelling the same as generalised linear modelling? If so, consistency needed throughout.

*No, they are different. A GLMM was used to model return rate and a GLM used to model. I’ve decided to remove the GLM of finishing time now I have included more detailed analysis of land cover types as I don’t think it contributed that much to the paper compared to the new additions.*

Line 110: how was “remoteness” of the event determined? This detail is needed in the methods.

*Details have been added to the Methods.*

You may want to refer to previous studies by Robert Smith and Paul Schneider that have looked specifically at parkrun locations in the England.
Smith, R. A., Schneider, P. P., Cosulich, R., Quirk, H., Bullas, A. M., Haake, S. J., &; Goyder, E. (2021). Socioeconomic inequalities in distance to and participation in a community-based running and walking activity: A longitudinal ecological study of parkrun 2010 to 2019. Health & place, 71, 102626-102626. doi:10.1016/j.healthplace.2021.102626
Schneider, P., Smith, R. A., Bullas, A. M., Quirk, H., Bayley, T., Haake, S. J., . . . Goyder, E. (2020). Multiple deprivation and geographic distance to community physical activity events — achieving equitable access to parkrun in England. Public health (London), 189, 48-53. doi:10.1016/j.puhe.2020.09.002

*Thanks for this. I’ve added citations of both papers in the Discussion when talking about directing the locations of future parkruns*

Results
Lines 129-130: “The mean travelling time to the next nearest event was 30 mins for those that returned and 33 for those that did not return.” How was travelling time calculated? This detail is needed in the methods.

*Much more details have been added.*

Line 158: Is this study only looking at “runners” or might some completion times be from those who walked (e.g., over 50-55mins might be consider walkers)? Be careful with terminology.

*Thanks for spotting that. This has been changed to participants. I’ve been through the manuscript and changed any references to runners where it really means a generic participant to participant.*

Line 168: “reasonable comparable to” 1) do you mean reasonably 2) it seems a bit vague to say “reasonably comparable” – is it comparable or not? Is this a meaningful comparison to make?

*That phrase has been removed*

Discussion
General comments about Discussion: In the introduction, parkrun is used here as an example of a ‘positive upstream’ initiative that can have an important role in the obesity epidemic. I would like to see the Discussion come back to this point – how do the findings support this claim and what are the wider implications?

*Yes, this is a good point. More discussion about this has been added.*

Line 181-182: “The wider gap in Scotland might be indicative of a larger gender gap in activity in Scotland”. Can you support this proposition with evidence/a citation?

*Yes, a citation has been added.*

Line 196: “lower” (travelling time) – might ‘shorter’ be a more appropriate word? I also want to know if this travelling time is by car, foot, as the crow flies or taking roads etc. Much more detail needed in Methods.

*Details have been added to the methods and yes I agree with the reviewer that shorter is a more appropriate word.*

Implications for parkrun
There is a focus on parkrun (understandably, given the data), but I wonder if the authors could demonstrate any wider implications of the findings for other community initiatives like parkrun? This is important for readers from countries that do not have parkrun events, and would increase the relevance/impact of the findings.

*Yes, I agree this has now been done.*

Line 227: the paper would benefit from introducing the parkrun practice in the Introduction – this could be part of the rationale for focussing on parkrun as a public health initiative.

*Yes, this has now added to the introduction.*

Limitations of the study
Line 236: “It is known that the gender gap in participation has been narrowing” – please specify, is this in the parkrun population? If so, provide a citation/reference.

*A citation has been added*
