# Supplementary material for: Predictors of successful return to parkrun for first-time adult participants in Scotland
Source: PLOS Glob Public Health. 2023 Aug 16;3(8):e0001786. doi: 10.1371/journal.pgph.0001786 (PMC10431652; doi:10.1371/journal.pgph.0001786)
Supplement: Attachment — Submitted filename: Response to reviewers 2.docx [file pgph.0001786.s002.docx]

Here are each of the comments from the reviewers and my response to them in italics. Once again I would to thank the reviewers for their contribution towards improving the manuscript further.

There is no overarching RQ but a series of RQs that while related necessitate the reader to make those connections. I would encourage you to consider identifying one over-arching RQ and then a series of sub-Qs (or perhaps even objectives?). I do wonder if a single focus upon geospatial features would make for a better paper?

*I have followed the reviewer’s suggestion of an overarching research question and a series of sub-questions. I believe this has added clarity to the purpose and scope of the manuscript.*

*The suggestion to just focus of geospatial features would remove the majority of the findings. The manuscript presented is data science. This is where you use, amongst other techniques, statistical models to extract knowledge and insights from very large noisy datasets that usually haven’t been collected for research purposes. In data science you use all the information available to develop the best model. The best models used here were objectively selected using mathematical calculations rather than being built based upon preconceived hypotheses. The models can be used to test hypotheses but don’t require them in their development. I have substantially restructured the Introduction to include a section reviewing the limited data science studies that have been carried on the parkrun results database. The aims have been restructured to make it clearer how this study builds upon what the previous data science parkrun studies have found. The overarching RQ of the paper is to use data science methods to extract novel information from the parkrun results database to help us better understand factors associated with whether or not new parkrunners return. Data science studies often incorporate a large component of hypothesis generation in addition to hypothesis testing The RQs have been rewritten to try to cover all the possible questions the study could address without making the list too cumbersome.*

You draw upon gender differences and barriers to exercise explicitly at the outset but then gender isn’t actually one of your questions / the focus of your paper (although it does then reappear so perhaps it does need to be included within the RQs?). I would encourage you to reconsider what you foreground in the introduction (see point 3)

*I have rewritten the RQs to include all the factors incorporated into the models including gender. The focus on genders in the Introduction is because there are well established addition barriers to women participating in sport and exercise and this has been the focus of much of the existing parkrun literature.*

I would encourage you to reconsider the structuring of the “Introduction” as at present there is no clear narrative through it that relates to and informs the overall purpose of your study. While I do recognise and appreciate that you have attempted to respond to the previous comments of the need to further recognise the extant literature, this has resulted in a somewhat amorphous section that is highly descriptive and needs greater synthesis. I do think all that you mention is relevant, it is just how it is structured and what is foregrounded / backgrounded that needs further reworking.

*I have substantially restructured the introduction including the addition of more signposting and section headings to try to create a much clearer narrative. I have also now explicitly discussed how this is a data science study which builds upon previous data science studies of the parkrun results dataset to help provide that clearer narrative.*

Would not the pandemic have affected return rates by changing behaviours, and would this potentially not be more important than the variables observed? For example, new runners may have, in that intervening time period moved up from being a new/novice runner and have moved onto longer runs / additional activities?

*That is entirely possible and something I’m currently exploring comparing data from before and after the pandemic in another set of models. There is a comment about this in relation to the relatively high proportion of returning parkrunners in Scotland where I have mentioned that this could be related to changes in behaviour as a result of lockdown and that this is something that is well worth following up. The addition of the pandemic won’t alter any of the findings in this paper although it could, as mentioned in the paper, provide an additional explanation for some of them.*

Discussion: I would encourage you to stick to what you have found out and try to explain these findings rather than trying to make unsubstantiated ‘perhaps’ assertions. Ensure that you focus upon your RQs in this section.

*I have reorganised and rewritten the Discussion to make it more focussed on the RQs and with clear section headings that match the broader RQs to add signposting.*

*I’ve been through the manuscript and altered the phrasing of sentences to make it clear that explanations of findings are hypotheses that are being generated for testing not claims about cause and effect. I’ve also added extra text to the limitations section noting the limitations of using correlational studies/data science for establishing cause and effect that make that clear across the entire manuscript. I have also rewritten the title to reflect the study is correlational rather than experimental.*

Minor points that you should also consider are:
1. Line 48 it is not clear why you are referring to “cycle routes” (as only an insider might recognise that parkruns are sometimes on cycle routes). Perhaps “traffic free routes” would be a better explanation at this point in the paper?

*Changed to traffic free routes as suggested.*

2. Line 64 “This is currently being promoted through the parkwalk” – what is? And walking what? – the route

*This has been clarified.*

3. Line 78 “In addition to the health benefits …” I think you mean physical health benefits as community, enhanced sense of self etc all relate to mental health – and therefore arguably are also ‘health benefits’ rather than ‘in addition to ..’

*Changed as suggested.*

4. Line 319 you refer to “moving water” but surely coastal parkruns would have far more moving water than the other water-based locations? This (among some other assertion within) brings me to question your assertions re freshwater throughout the paper as the characteristics of it that you assert are very similar, if not more pertinent, for coastal locations as well. I think you need greater clarity here therefore in distinguishing these.

*I have added discussion of why freshwater and saltwater routes might have different impacts upon return rates.*

5. Lines 363-364, you suggest that more returners are female but I didn’t see this within the data that you presented

*The manuscript provided the sample sizes and the return rates for both genders so they could be calculated. I have now also added the actual number of returners to the results.*

Given that much of the manuscript has been revised, I have some additional comments to make. These are just minor:
Line 75: should run be 'runs'

*Thanks for spotting that, changed as suggested.*

Line 98/99: 'performance of the parkrun population was falling' didn't quite sit right with me. Consider rephrasing. "the average finish time is getting longer" (again, I'm careful not to use the word slower, as I think that's a bit degrading!)

*Performance is the correct term here as the cited study modelled age graded performance scores rather than finishing times.*

Line 105: is there scope to briefly add what some of the major barriers to participation were here?

*Yes there is and this has now been added.*

Line 179: KML format- does this need writing in full?

*I have now given the full name.*

Line 290-292: repetition of 'relatively slow' in this sentence. Is there any evidence/citation to support this claim of slower participants feeling like they're holding up the event? (I'm wondering if any of the barriers to parkrun research has this evidence?)

*Feeling like you’re going to be too slow was found to be a key main barrier to participation in the Reece et al 2022 study. I’ve added in more discussion and a citation.*

line 343: check consistency of using hyphen in 'first-time' throughout manuscript

*That’s for spotting that. Hyphen now consistently used.*
