# Supplementary material for: Predictors of successful return to parkrun for first-time adult participants in Scotland
Source: PLOS Glob Public Health. 2023 Aug 16;3(8):e0001786. doi: 10.1371/journal.pgph.0001786 (PMC10431652; doi:10.1371/journal.pgph.0001786)
Supplement: Attachment — Submitted filename: Third response to reviewers.docx [file pgph.0001786.s003.docx]

Editors Suggestion

Having reviewed the outcomes of the rounds of reviews submitted, it is believed that the manuscript would greatly benefit from a title modification from what it is currently to "Predictors of successful return to Parkrun among first-time adults in Scotland" considering that the short title is "Factors Associated With Return Rates to parkrun" and harmonizes appropriately with the suggested revised title and the data analysis in the manuscript and findings. With this revision the manuscript is well poised to communicate adequately with potential readers. If this suggestion is accepted, kindly replace "novel characteristics" and modify the statement of the aim to align appropriately with the revised title.

*This is an excellent suggested revision to the title. I changed the title as suggested and made addition revisions to make the text of the manuscript more consistent with the title.*


Reviewer #1: Thank you very much indeed for the opportunity to further re-review your manuscript. I appreciate you responding to many of the comments in this revised manuscript. In particular, thank you for ensuring that the study aim, and associated questions, are now clearly stated and that the narrative through the paper is much more apparent. Thank you.

My suggestions are as follows:
1. Your work would have greater initial impact, and understanding, if you direct the reader towards parkrun earlier in your initial statements around exercise, by drawing upon parkrun-relevant examples – for example that exercising with others can be really important … rather than e.g. provision of off road routeways!

*The reference to traffic-free routes is one of the key positive upstream factors that contributes to increases in activity and it is relevant to parkrun as one of the requirements for a parkrun route is that it is traffic-free so I’ve revised this section as suggested by the referee by mentioning parkrun earlier and the social benefits of parkrun but have also retained mention of traffic-free routes as an important positive upstream factor that is an integral part of parkrun. I agree with the reviewer that this change to direct readers towards parkrun earlier is beneficial.*

2. That many other quants studies have taken place isn’t in itself a reason to undertake the work! Are you better able to justify the study?

*I have now added in additional justification for the approach taken. N.B. The study isn’t justified on the basis of there being many other quants studies. The study is justified on the basis of filling key knowledge gaps. To date most studies of parkrun have actually used a qualitative not a quantitative approach, which is discussed. There is a section in the introduction on how a data science approach could aid filling knowledge gaps with existing datasets but that very few studies have used this approach.*

3. Some of the variables that you state within the RQs aren’t entirely evident in your preceding literature review

*Several of the variables have only been considered by a single previous study whether in the context of parkrun or other mass participation events. I have added an additional section to the aims referring to that study in more detail and included some additional justification for investigating these factors further.*

4. As previously, research has shown that the pandemic has significantly changed individuals’ behaviours, and especially towards exercise. Therefore, I would encourage you to consider would not the pandemic have affected return rates by changing behaviours, and would this potentially not be more important than the variables observed? For example, new runners may have, in that intervening time period moved up from being a new/novice runner and have moved onto longer runs / additional activities? You acknowledge this in your comments but I would have expect to see some recognition of this in the “limitations”

*Several studies show that the pandemic has indeed changed behaviour with respect to exercise but the consistent trend across these studies is a substantial reduction in exercise, so there is no evidence supporting the reviewer’s suggestion. Indeed the data suggest the opposite so I think reference to this would be too speculative. There is already a sizable section in the limitations section on how the pandemic could have impacted the findings which I have now expanded to make the potential impacts of the pandemic on the findings clearer.*
